# Evaluation of the results of the patients who underwent plasmapheresis in the pediatric intensive care unit

**DOI:** 10.55730/1300-0144.5817

**Published:** 2024-03-04

**Authors:** Mehmet Nur TALAY, Özhan ORHAN, Murat KANĞIN, Eşe Eda TURANLI, Mehmet Nuri ÖZBEK

**Affiliations:** 1 Department of Pediatric, Faculty of Medicine, Mardin Artuklu University, Mardin, Turkiye; 2 Department of Pediatric Intensive Care, Faculty of Medicine, İstanbul Medipol University, İstanbul, Turkiye; 3 Department of Pediatric Intensive Care, Gazi Yaşargil Training and Research Hospital, Diyarbakır, Turkiye; 4 Department of Pediatric Endocrinology, Faculty of Medicine, Mardin Artuklu University, Mardin, Turkiye

**Keywords:** Therapeutic plasma exchange, pediatric intensive care unit, mortality, multisystem inflammatory syndrome

## Abstract

**Background/aim:**

Therapeutic plasma exchange (TPE) is an extracorporeal treatment method that removes large molecular weight substances from plasma. In our study, we aimed to retrospectively examine the indications and procedural methods of the patients who had undergone TPE, and the complications that occurred during the procedure.

**Materials and methods:**

Forty-one patients who were monitored in thePICU of Gazi Yaşargil Training and Research Hospital and had indications for TPE between 2017 and 2021 were included in the study. Laboratory parameters were checked before and after the TPE procedure. In addition to these, patients’ diagnosis, weight, type of procedure and type of device, where the procedure was performed, duration of the procedure, amount of blood and plasma processed, complications, number of procedures, and death during the procedure or independent of the procedure were evaluated.

**Results:**

The median age was 93.0 (14.0–167.0) months. Hemolytic uremic syndrome (HUS) was the most common TPE indication with nine patients. The most common complication related to TPE was fever (11 patients), while no complication was observed in 18 patients.

When laboratory results were evaluated according to American Society for Apheresis (ASFA) categories, a significant improvement was observed in the values of platelet, AST, ALT, LDH, urea, and creatinine in ASFA1 after TPE. No significant improvement was observed in ASFA2 (p > 0.05). In ASFA3, a significant improvement was observed in INR, AST, ALT, LDH, total bilirubin, creatinine, pH, and lactate values after TPE (p < 0.05). Five patients died from ASFA1, one from ASFA2, and three patients from ASFA3.

**Conclusion:**

Since significant adjustments are observed in clinical and laboratory values in sepsis-MOF, which is in the ASFA3 category, we believe that it should be evaluated in the ASFA2 or ASFA1 category in the early treatment of these diseases. In addition, we think that MIS-C cases, which have not been in any category according to ASFA, should be included in the ASFA2 or ASFA3 category, considering our TPE results.

## 1. Introduction

Therapeutic plasma exchange (TPE) is an extracorporeal treatment method that removes large molecular weight substances from plasma. The patient’s blood is taken into the extracorporeal system, and the plasma and cellular elements of the blood are separated from each other. Fresh frozen plasma (FFP), albumin, or crystalloid-colloid combinations are substituted for the separated plasma. During the procedure, it is combined with the cell-rich part of the blood and given back to the patient [1].

TPE is performed using one of two methods: centrifugal separation or membrane-based (filtration) separation, with neither method being superior to the other [2]. Target molecular properties such as large molecular weight increase the effectiveness of TPE. This feature makes TPE superior to other extracorporeal therapeutic modalities. Features such as slow formation rate, low turnover, low volume of distribution, and a defined etiological agent may also constitute target molecule properties suitable for TPE [3].

According to the diagnoses, TPE is performed based on the category variables determined by the American Society for Apheresis (ASFA). The ASFA category variables were updated in 2019 and are subject to change every 3 years (Table 1). According to these categories, diseases in which TPE is the primary treatment method fall in ASFA1, diseases in which TPE is the second-line treatment method, alone or together with other treatment methods, in ASFA2, diseases in which the optimum role of TPE cannot be determined in ASFA3, and diseases in which apheresis is shown or suggested to be ineffective or harmful according to published evidence in ASFA4, that is, apheresis treatment applications in such situations should only be performed under approved research protocols [2].

When TPE indications were examined in pediatric intensive care units (PICU), neurological diseases were in the first place in previous years. However, it is now more frequently applied in cases of sepsis-related multi-organ failure (sepsis-MOF) [4,5]. The ASFA criteria classify sepsis-MOF cases under category 3 (diseases for which the optimal role cannot be definitively determined). It has been found that TPE procedures are performed more frequently in patients with a diagnosis of sepsis-MOF [2,6,7].

Liver failure is a rare but fatal clinical condition seen in the pediatric age group. It manifests itself with clinical and laboratory findings such as hepatic encephalopathy, hepatic cardiopathy, hepatorenal syndrome, coagulopathy, especially caused by substances such as toxins, aromatic amino acids, ammonia, endotoxins, and indoles. Therapeutic plasma exchange for liver failure is done as a bridge therapy to save time for liver transplantation or for therapeutic purposes that facilitate complete recovery. TPE is among the first-line treatments for fulminant liver failure, especially in cases of life-threatening coagulopathy and bleeding [8].

Plasma exchange is effective in the treatment of disease by eliminating circulating antibodies associated with the disease in the plasma. In autoimmune hemolytic anemia, plasmapheresis is recommended as a third-line treatment in patients who urgently need transfusion until the effects of immunosuppressive treatments are observed. It is also recommended for patients who fail immunosuppressive therapy and splenectomy, and whose disease recurs [9,10].

In May 2020, a national health guide was published by the Centres for Disease Control and Prevention (CDC) to identify patient groups meeting the criteria for multisystem inflammatory syndrome in children (MIS-C). According to this definition, MIS-C occurs approximately 4–6 weeks after acute SARS-CoV-2 infection. It is a disease that develops with an excessive immune response triggered by infection, rather than an acute manifestation of viral disease^1^. Intravenous immunoglobulin (IVIG), pulse steroid, and plasmapheresis are effective in its treatment [11].

The complication rate associated with the TPE procedure ranges between 0.2% and 0.025%. Serious life-threatening complications are collected in two groups. The first group comprises catheter-related complications, catheter-related thrombosis, hemorrhage, infection, pneumothorax, and mechanical complications. The second group includes procedure-related complications, hypotension requiring catecholamine, arrhythmia requiring drug therapy, and hemolysis. Nonlife-threatening complications are hypotension, fever, urticaria, hypercalcemic findings, itching, tachycardia, nausea, vomiting, abdominal pain, anxiety, and muscle cramps that do not require catecholamines [12].

In our study, we aimed to retrospectively examine the indications and procedural methods of the patients who had undergone TPE, and the complications that occurred during the procedure.

## 2. Materials and methods

Forty-one patients who were monitored in thePICU of Gazi Yaşargil Training and Research Hospital and had indications for TPE between January 1, 2017, and October 27, 2021, were included in the study. Demographic data and pre- and postprocedural laboratory parameters of the patients were recorded. The effects of the applied procedure on the effectiveness, safety, and life span were examined. Laboratory parameters (biochemistry, hemogram, coagulation, and blood gas) were checked before and after the TPE procedure. In addition to these, patients’ diagnosis, weight, type of procedure and type of device, where the procedure was performed, duration of the procedure, amount of blood and plasma processed, complications, number of procedures, and death during the procedure or independent of the procedure were evaluated. Patients who were in another study and were older than 18 years were excluded from the study.

The TPE procedure was applied at the patient’s bedside in the PICU by placing a central venous catheter providing adequate blood flow. In all cases, the central venous route was utilized, and the peripheral route was not employed. The total number of TPE procedures for each patient and the session intervals were determined based on the clinical and laboratory response of the patients. Total plasma volume was found using the formula: Total blood volume × (1-hematocrit). The amount of the replacement fluid was calculated as 1 or 1.5 times the plasma volume calculated according to the clinical condition of the patients. FFP was used in therapeutic plasmapheresis procedures. TPE procedures were performed with a Prismaflex 2015 model automatic apheresis device using venous access.

The data of the patients were shown in the study as descriptive data, and as n, % values, and median interquartile range (25–75 percentile values) in categorical data. The normality analysis of the data was done with the Shapiro–Wilk test. Wilcoxon analysis was performed to compare the values before and after plasmapheresis. The statistical significance level in the analyses was accepted as p < 0.05.

Approval for the study was obtained from the ethics committee of Diyarbakır Gazi Yaşargil Training and Research Hospital on November 26, 2021, with approval number 935.

## 3. Results

A total of 41 patients, 23 of whom were male, underwent TPE and were included in the study. Among them, three patients were transferred to our unit specifically for TPE procedures while they were being monitored in other centers. The median age was 93.0 (14.0–167.0) months. Hemolytic uremic syndrome (HUS) was the most common TPE indication with nine patients. Eight of the patients were hospitalized in the PICU with the diagnosis of Guillain–Barré syndrome, eight with MIS-C, five with liver failure, three with sepsis-MOF, two with acute disseminated encephalomyelitis, two with autoimmune hemolytic anemia, two with autoimmune encephalitis, one with hypertriglyceridemia, and one with drug intoxication. When the ASFA classification of the patients was examined, it was determined that 19 of them were category 1, 3 were category 2, and 11 were category 3 (Table 2). Eight of our patients were not included in the ASFA category because of MIS-C.

A total of 119 sessions of TPE were performed. Standard TPE was applied to 28 (56%) patients at a one-to-one ratio, and to 13 (26%) patients at a one-to-one-half ratio. The mean plasma volume given during the procedure was 1514.39 ± 1111.26 mL. Twenty-seven of the patients who underwent TPE responded to the treatment and were transferred to the service, 23 received mechanical ventilator (MV) support, and 5 patients were transferred to their own center after the TPE procedure, and 9 patients died. Ten of the patients did not receive additional treatment, 19 received IVIG, 6 received hemodiafiltration, 5 received peritoneal dialysis, and 1 (2.4%) received pulse steroids. The most common complication related to TPE was fever (11 patients), while no complication was observed in 18 patients. None of our patients died due to the TPE procedure (Table 3).

The comparison of pre- and postprocedural blood values of the patients who underwent TPE is presented in Table 4. Platelet (p = 0.035) and active partial thromboplastin time test (aPTT) (p < 0.001) values of the patients increased significantly after the TPE procedure. The aPTT increase may be due to the use of heparin anticoagulation during the TPE procedure. Prothrombin time test (PT) (p = 0.01), international normalized ratio (INR) (p = 0.001), aspartate aminotransferase (AST) (p < 0.001), alanine aminotransferase (ALT) (p < 0.001), lactate dehydrogenase (LDH) (p < 0.001), total bilirubin (p = 0.032), urea (p < 0.001), and creatinine (p < 0.001) values of patients decreased significantly after TPE.

When laboratory results were evaluated according to ASFA categories, a significant improvement was observed in the values of platelet, AST, ALT, LDH, urea, and creatinine in ASFA1 after TPE (p < 0.05). No significant improvement was observed in ASFA2 (p > 0.05). In ASFA3, a significant improvement was observed in INR, AST, ALT, LDH, total bilirubin, creatinine, pH, and lactate values after TPE (p < 0.05). Similar to the patients in the ASFA category, the MIS-C patients showed significant improvement in neutrophil-lymphocyte ratio (NLR), thrombocyte, PT, INR, AST, ALT, LDH, urea, creatinine, pH, and lactate values (p < 0.05) (Table5).

Two of our patients who underwent plasmapheresis due to liver failure were referred to an advanced center due to the need for liver transplantation. We transferred our three patients whose plasmapheresis procedure was completed to the center where they were followed up after the procedure.

Five patients from ASFA1, one from ASFA2, and three patients from ASFA3 died. Seven of our patients died 48–72 hours after the TPE procedure, and two died after the 7th day.

## 4. Discussion

Eleven of the patients in our study were in the ASFA3 category. The ASFA3 category includes sepsis-MOF, liver failure, IgA nephropathy, focal segmental glomerulosclerosis, and hemophagocytic lymphohistiocytosis. The majority of our patients in ASFA3, in which we applied TPE, consisted of hepatic failure and sepsis-MOF cases. We lost one patient from each of these two disease groups, and the reason for these losses was not the TPE procedure. In cases in the ASFA3 category, laboratory and clinical improvement was better than ASFA1 and ASFA2 (Table 5). It is not clear whether cases in the ASFA3 category will benefit from the TPE procedure. Considering our own cases, we observed that the results of the procedure were at least as effective as those of ASFA1 and ASFA2 cases. In fact, Emeksiz et al. reported in their study that 76.2% of the patients who underwent TPE had ASFA category 3 disease. Again, in the same study, they applied TPE to 23 (53.5%) patients due to sepsis-MOF and they discharged 19 (82.6%) of the patients [6]. Similarly, in our study, we applied TPE to three patients due to sepsis-MOF and discharged two (66.7%) patients.

Keskin et al. emphasized that the combination of IVIG, steroid, and plasmapheresis could be lifesaving in a patient diagnosed with MIS-C and having cardiac involvement [11]. In our study, among the 8 patients diagnosed with MIS-C with cardiac and/or cerebral involvement, clinical improvement was achieved in 7 patients (87.5%) who were treated with both IVIG and plasmapheresis, and one patient (12.5%) died 48 h after plasmapheresis. Laboratory improvement in MIS-C cases was similar to that in the ASFA3 category. We performed plasmapheresis in MIS-C disease because of thrombotic microangiopathy secondary to inflammation and multiorgan involvement. The diagnosis of MIS-C was not yet in any category according to ASFA at the time we authored our study. We observed that the results of the procedure were as effective as the ASFA2 and ASFA3 cases in our MIS-C patients who underwent TPE. In the light of the data we obtained from our study, it was evaluated that it would be appropriate to include MIS-C disease in the ASFA 2 or 3 category.

The use of TPE in the treatment of immune-mediated renal diseases is increasing. It is suggested that the early implementation of TPE in adult patients will improve the prognosis of HUS [13]. In a study reporting TPE application in children, 2 of 43 (4.6%) patients were diagnosed with hemolytic uremic syndrome (9). In our study group, TPE was applied to 9 (22%) patients with the most common diagnosis of atypical hemolytic uremic syndrome. Three of these patients (33.3%) died due to sepsis and pneumonia in their subsequent clinical follow-up.

TPE treatment applied in patients diagnosed with Guillain-Barré syndrome (GBS) accelerates the recovery in motor nerves and reduces the duration of mechanical ventilation [4,6]. In various studies, between 9.4% and 46.4% of the cases who underwent TPE procedure were patients with a diagnosis of GBS [4,6,14]. Eight of the patients in our study group underwent TPE due to GBS. Clinical improvement was achieved in seven of these patients (87.5%), tracheostomy was performed in two of our patients, and one of them died due to pneumonia 11 days after the plasmapheresis procedure.

Larsen et al. showed in their study of adults that plasmapheresis was effective in the treatment of patients with acute liver failure without the need for liver transplantation [15]. In another study, it was reported that TPE can be applied in cases where there is no response to other treatments in hepatic failure due to sepsis [16]. In our study, two of the 5 patients who underwent TPE due to liver failure were discharged, two were transferred to the transplantation center, and one patient died. The satisfactory response of the patients to the treatment was evaluated in line with this result.

In our study, TPE was performed in one patient for intoxication due to carbamazepine intake, and in one patient for pancreatitis due to hypertriglyceridemia. Consistent with the literature, clinical improvement was observed in both cases [17–19].

Studies have shown that TPE may be beneficial in patients with autoimmune hemolytic anemia (AIHA) who do not respond to steroid and intravenous immunoglobulin (IVIG) treatment [10,20]. In our study, we applied TPE to 2 cases due to AIHA. In the acute period, clinical improvement was achieved in both patients, and they were discharged with steroid treatment.

Studies have reported that the complication rate of TPE in pediatric patients is 1%–40% [21]. In a study by Tolunay et al., the most common complication was hypotension with a rate of 29.2% (12/41). Allergic reactions such as urticaria and fever were 9.7% (4/41) and hypertension 4.8% (2/41). Again, in the same study, they did not observe any catheter-related complications, and none of their patients died due to the plasmapheresis procedure [4]. In our study, complications were observed in 23 (56%) patients. Allergic reactions such as urticaria and fever were the most common complications in 34.1% (16/41), while no catheter-related complications were observed.

Studies have reported mortality due to TPE to be 0.05%. However, it was also noted that these patients died of underlying diseases [22]. In our study, none of the patients died due to the TPE procedure.

The limitation of our study is that it is single-center, and the sample size is not large enough to make a healthy evaluation.

As a result, we can say that TPE, used in addition to standard treatments in underlying autoimmune diseases, liver failure, and sepsis-MOF, increases survival rates and is beneficial for prognosis. The experience with plasmapheresis varies from center to center. Since significant adjustments in clinical and laboratory values are observed in sepsis-MOF, which is in the ASFA3 category, we believe that it should be evaluated in the ASFA2 or ASFA1 category in the early treatment of these diseases. In addition, we think that MIS-C cases, which have not been in any category according to ASFA, should be included in the ASFA2 or ASFA3 category, considering our TPE results.

## Figures and Tables

**Table 1:** ASFA categories by diagnosis.

Diagnosis	ASFA 2019 category
**Hemolytic uremic syndrome**	I
**Guillain–Barré syndrome**	I
**Autoimmune encephalitis**	I
**Acute disseminated encephalomyelitis**	II
**Intoxications**	II–III
**Sepsis, multiple organ failure**	III
**Liver failure**	III
**IgA nephropathy, focal segmental glomerulosclerosis**	III
**Hemophagocytic lymphohistiocytosis**	III
**ASFA**; American Society for Apheresis	

**Category I:** Diseases for which therapeutic plasma exchange (TPE) alone is the primary treatment method.

**Category II:** Diseases in which TPE is the second-line treatment method, alone or together with other treatment methods.

**Category III:** Diseases in which the optimum role of TPE cannot be determined.

**Category IV:** Diseases for which apheresis is shown or suggested to be ineffective or harmful based on published evidence. Apheresis treatment practices in this situation should only be performed under approved research protocols.

**Table 2:** ASFA categories and diagnoses of the patients.

	n (%)	Diagnosis	n (%)	Exitus n(%)	Exitus n(%)
**ASFA category 1**	**19 (46.3)**	**Hemolytic uremic syndrome**	9 (22.0)	3 (7.3)	5 (12.2)
		**Guillain–Barré syndrome**	8 (19.5)	1 (2.4)	
		**Autoimmune encephalitis**	2 (4.9)	1 (2.4)	
**ASFA category 2**	**3 (7.3)**	**Acute disseminated encephalomyelitis**	2 (4.9)	1 (2.4)	1 (2.4)
		**Intoxications**	1 (2.4)	-	
**ASFA category 3**	**11 (26.8)**	**Liver failure**	5 (12.2)	1 (2.4)	3 (7.3)
		**Sepsis, multiple organ failure**	3 (7.3)	1 (2.4)	
		**Autoimmune hemolytic anemia**	2 (4.9)	-	
		**Hypertriglyceridemia**	1 (2.4)	-	
**MIS-C**	**8 (19.5)**			1 (2.4)	1 (2.4)

**ASFA**, American Society for Apheresis; **MIS-C**, Multisystem inflammatory syndrome in children.

**Table 3:** All characteristics of the patients.

		Person	%
**Sex**	**Female**	18	43.9
	**Male**	23	56.1
**Age (months), Mean (IQR)**		93.0 (14.0–167.0)	
**Body surface area, Mean (IQR)**		0.9 (.4–1.6)	
**Application period (hours), Mean (IQR)**		6.0 (4.0–9.0)	
**Number of applications, Mean (IQR)**		2.0 (2.0–4.0)	
**Glasgow Coma Scale, Mean (IQR)**		12.0 (10.0–15.0)	
**Pediatric Risk of Mortality Score, Mean (IQR)**		15.0 (11.0–19.0)	
**Exitus**		11	26.8
**Mechanical ventilator support**		23	56.1
**Time on mechanical ventilator (hours), Mean (IQR)**		11.0 (6.0–22.0)	
**ICU length of stay (days), Mean (IQR)**		13.0 (7.0–24.0)	
**Length of stay in hospital (days), Mean (IQR)**		18.0 (12.0–39.0)	
**Complication**	**High body temperature**	11	26.8
	**Rash, allergic reaction**	6	14.6
	**Hypotension**	3	7.3
	**Hypertension**	2	4.9
	**Hypovolemia**	1	2.4
	**Without complications**	18	43.9
**Additional treatment**	**Intravenous immunoglobulin**	19	46.3
	**Hemodiafiltration**	6	14.6
	**Peritoneal dialysis**	5	12.2
	**Steroid**	1	2.4
	**No additional treatment**	10	24.4

**Table 4:** Comparison of blood values before and after plasmapheresis.

	Before	After	p*
	Mean (IQR)	Mean (IQR)	
**Neutrophil/lymphocyte ratio**	3.7 (0.24–28.09)	2.84 (0.12–68.11)	0.12
**Hemoglobin**	10.6 (0.9–16.14)	9.4 (5.2–16.3)	0.52
**Hematocrit**	32.0 (2.3–50.2)	29.5 (16–48)	0.75
**Platelet**	187000.0 (17000–430000)	212000.0 (53000–505000)	0.035
**Prothrombin time**	14.9 (11–43)	13.2 (10–40)	0.015
**aPTT**	27.7 (17–94)	25.8 (13.3–42.2)	0.52
**INR**	1.31 (1.0–3.9)	1.23 (0.9–1.7)	0.009
**Calcium**	8.26 (5.45–10.40)	8.6 (5.47–10.7)	0.76
**AST**	103 (12.3–4202)	37.3 (11.3–1675)	0.000
**ALT**	221.1 (10–4113)	29.4 (9–1134)	0.000
**LDH**	543 (134–13330)	374 (122–1619)	0.000
**Sodium**	137 (124–165)	137.0 (130–151)	1.0
**Potassium**	3.9 (2.15–5.39)	3.81(2.73–5.25)	0.874
**Albumin**	32 (13–55)	31 (22–46)	0.735
**Total bilirubin**	0.62 (0.2–19.28)	0.60 (0.14–6.91)	0.337
**Indirect bilirubin**	0.9 (0.32–10.84)	0.28 (0.04–3.95)	1.0
**Urea**	57 (9–222)	30 (14–148)	0.012
**Creatinine**	0.9 (0.32–6.76)	0.57 (0.28–5.5)	0.000
**pH**	7.32(7.0–7.56)	7.38(7.29–7.51)	0.061

**aPTT**, active partial thromboplastin time test; **INR**, international normalized ratio; **AST**, aspartate aminotransferase; **ALT**, alanine aminotransferaz; **LDH**, lactate dehydrogenase; **pH**, power of hydrogen.

* Wilcoxon analysis was applied.

**Table 5:** Comparison of blood values before and after plasmapheresis in ASFA categories.

ASFA categories		ASFA 1		ASFA 2		ASFA 3		MIS-C	
		Mean (IQR)	p^*^	Mean (IQR)	p^*^	Mean (IQR)	p^*^	Mean (IQR)	p^*^
**Neutrophil/lymphocyte ratio**	Before	3.78(0.35–28.09)	0.658	3.89(1.23–9.32)	0.593	1.58(0.24–11.05)	0.48	18.67(3.09–22.32)	0.012
	After	3.07(0.12–68.11)		3.21(1.38–13.98)		1.56(0.15–9.71)		3.38(0.8–11.73)	
**Hematocrit**	Before	27(17.7–50.2)	1.00	32.4(32–40.3)	0.655	31.30(2.30–43.10)	0.86	31.5(18–37.6)	0.76
	After	28.2(16–48)		33.5(31–40.3)		26.9(22–41.5)		30.25(20–38.8)	
**Platelet**	Before	89(26–43)	0.067	227(187–228)	0.109	228(20–395)	0.37	177.5(17–277)	0.02
	After	212(53–493)		243(194–248)		144(65–310)		239(152–505)	
**Prothrombin time**	Before	12.4(10.8–21)	0.623	14.9(14–14.9)	0.285	18 (11–34)	0.09	17.35(12–43)	0.02
	After	12.6(10–16.4)		17.8(13.2–18.2)		14.5(12–40)		12.95(10–16)	
**INR**	Before	1.15(0.95–2.73)	0.642	1.3(1.3–1.37)	0.593	1.54 (1.1–2.9)	0.01	1.62(1.1–3.9)	0.02
	After	1.16(0.9–1.54)		1.32(1.22–1.6)		1.24(1.0–1.7)		1.205(0.9–1.5)	
**Calcium**	Before	8.26(6.34–10.4)	0.295	8.8(8.4–9.1)	0.109	8.12 (5.45–9.9)	0.25	8.06(5.6–9.0)	0.141
	After	8.6(7.1–10.7)		8.1(7.7–8.59)		8.73(5.47–10.26)		8.7(8.1–9.5)	
**AST**	Before	102(12.3–2086)	0.001	30(17–638)	0.593	949.7 (25–4202)	0.003	79.85(21.1–4202)	0.04
	After	36(18.3–317)		29(25.3–403)		41(11.3–1675)		47.15(22.30–182)	
**ALT**	Before	47(10.5–2716)	0.002	19(10–537)	1.000	990.5(16–4113)	0.004	311.45(143.4–3799)	0.012
	After	21(9–74)		28.2(17–261)		83.1(13–1134)		42(9.6–342)	
**LDH**	Before	496(154–3775)	0.001	194(183–1462)	0.593	1456(419–6978)	0.003	391.5(134–13330)	0.03
	After	330(155–876)		294(122–681)		520(124–1619)		225.5(164–781)	
**Sodium**	Before	133(124–144)	0.211	137(131–141)	0.285	142 (125–165)	0.13	136(131–158)	0.778
	After	138(130–144)		136(135–143)		137(133–146)		137(132–151)	
**Potassium**	Before	4.03(2.4–5.03)	0.828	3.5(3.3–3.9)	0.109	4.09(2.15–5.39)	0.66	3.18(2.7–4.49)	0.401
	After	3.81(2.76–5.25)		4.3(3.4–4.75)		3.66(3.01–4.69)		3.85(2.73–5.1)	
**Albumin**	Before	28(19–55)	0.641	34(32–37)	0.157	34(13–41)	0.64	28(18–33)	0.268
	After	32(24–39)		33(32–36)		31(26–46)		29(22–35)	
**Total bilirubin**	Before	0.51(0.28–1.1)	0.760	0.53(0.4–0.86)	0.655	9.58(0.38–19.28)	0.02	0.44(0.2–1.3)	0.89
	After	0.54(0.21–1.31)		0.59(0.4–0.59)		1.2(0.45–6.91)		0.49(0.14–1.0)	
**İ** **ndirect bilirubin**	Before	0.3(0.14–0.7)	0.632	0.26(0.2–0.55)	0.180	1.14(0.04–10.84)	0.29	0.19(0.1–0.3)	0.917
	After	0.28(0.1–0.79)		0.2(0.18–0.22)		0.66(0.22–3.95)		0.2 (0.04–0.4)	
**Urea**	Before	54.5(9–222)	0.010	38(36–58)	1.000	24(9.1–147)	0.06	107.3(84–156)	0.012
	After	32(0.14–148)		37(31–51)		21(5.2–98)		33.5(13–101)	
**Creatinine**	Before	0.82(0.34–6.76)	0.000	0.77(0.44–1.1)	0.180	0.46(0.32–4.97)	0.03	1,61(0.82–5.4)	0.017
	After	0.59(0.4–4.07)		0.59(0.44–0.86)		0.44(0.28–2.17)		0.62(0.37–5.5)	
**pH**	Before	7.39(7.2–7.5)	0.841	7.42(7.3–7.42)	q1.000	7.3(7–7.56)	0.04	7.29(7.17–7.39)	0.012
	After	7.37(7.29–7.5119)		7.33(7.32–7.48)		7.0(7.36–7.49)		7.38(7.35–7.48)	
**Lactate**	Before	1.7(0.5–4.11)	0.702	1.61(1.55–3.9)	0.593	3.4(1.21–25.28)	0.03	6.5(3.6–7.8)	0.012
	After	2.18(0.74–3.84)		1.65(1.1–2.12)		1.97(1.16–3.84)		1.98(1.1–2.95)	

**ASFA**, American Society for Apheresis; **MIS-C**, multisystem inflammatory syndrome in children; **INR**, international normalized ratio; **AST**, aspartate aminotransferase; **ALT**, alanine aminotransferase; **LDH**, lactate dehydrogenase; **pH**, power of hydrogen.

## Footnotes

1 Centers for Disease Control and Prevention Health Alert Network (HAN)Multisystem inflammatory syndrome in children (MIS-C) associated with coronavirus disease 2019 (COVID-19). Available at https://emergency.cdc.gov/han/2020/han00432.asp accessed on 5 November 2020.
